# Circulating pyruvate is a potent prognostic marker for critical COVID-19 outcomes

**DOI:** 10.3389/fimmu.2022.912579

**Published:** 2022-09-14

**Authors:** Victòria Ceperuelo-Mallafré, Laia Reverté, Joaquim Peraire, Ana Madeira, Elsa Maymó-Masip, Miguel López-Dupla, Alicia Gutierrez-Valencia, Ezequiel Ruiz-Mateos, Maria José Buzón, Rosa Jorba, Joan Vendrell, Teresa Auguet, Montserrat Olona, Francesc Vidal, Anna Rull, Sonia Fernández-Veledo

**Affiliations:** ^1^Universitat Rovira i Virgili (URV), Tarragona, Spain; ^2^Institut Investigació Sanitària Pere Virgili (IISPV), Tarragona, Spain; ^3^CIBER de Diabetes y Enfermedades Metaboílicas Asociadas (CIBERDEM)-Instituto de Salud Carlos III, Madrid, Spain; ^4^CIBER Enfermedades Infecciosas (CIBERINFEC)-Instituto de Salud Carlos III, Madrid, Spain; ^5^Hospital Universitari de Tarragona Joan XXIII (HJ23), Tarragona, Spain; ^6^Clinical Unit of Infectious Diseases, Microbiology and Preventive Medicine, Institute of Biomedicine of Seville (IBiS), Virgen del Rocío University Hospital, Consejo Superior de Investigaciones Científicas (CSIC), University of Seville, Seville, Spain; ^7^Infectious Diseases Department, Vall d’Hebron Institute of Research (VHIR), Hospital Universitari Vall d’Hebron, Universitat Autònoma de Barcelona, (VHIR) Task Force COVID-19, Barcelona, Spain

**Keywords:** COVID-19, energy-related metabolites, fluorometric quantification, pyruvate, semi-targeted metebolomics

## Abstract

**Background:**

Coronavirus-19 (COVID-19) disease is driven by an unchecked immune response to the severe acute respiratory syndrome coronavirus 2 (SARS-CoV-2) virus which alters host mitochondrial-associated mechanisms. Compromised mitochondrial health results in abnormal reprogramming of glucose metabolism, which can disrupt extracellular signalling. We hypothesized that examining mitochondrial energy-related signalling metabolites implicated in host immune response to SARS-CoV-2 infection would provide potential biomarkers for predicting the risk of severe COVID-19 illness.

**Methods:**

We used a semi-targeted serum metabolomics approach in 273 patients with different severity grades of COVID-19 recruited at the acute phase of the infection to determine the relative abundance of tricarboxylic acid (Krebs) cycle-related metabolites with known extracellular signaling properties (pyruvate, lactate, succinate and α-ketoglutarate). Abundance levels of energy-related metabolites were evaluated in a validation cohort (n=398) using quantitative fluorimetric assays.

**Results:**

Increased levels of four energy-related metabolites (pyruvate, lactate, a-ketoglutarate and succinate) were found in critically ill COVID-19 patients using semi-targeted and targeted approaches (p<0.05). The combined strategy proposed herein enabled us to establish that circulating pyruvate levels (p<0.001) together with body mass index (p=0.025), C-reactive protein (p=0.039), D-Dimer (p<0.001) and creatinine (p=0.043) levels, are independent predictors of critical COVID-19. Furthermore, classification and regression tree (CART) analysis provided a cut-off value of pyruvate in serum (24.54 µM; p<0.001) as an early criterion to accurately classify patients with critical outcomes.

**Conclusion:**

Our findings support the link between COVID-19 pathogenesis and immunometabolic dysregulation, and show that fluorometric quantification of circulating pyruvate is a cost-effective clinical decision support tool to improve patient stratification and prognosis prediction.

## Introduction

The continuing pandemic of coronavirus disease-2019 (COVID-19) remains a challenge to healthcare systems worldwide (1). COVID-19 is a complex multisystemic disorder involving immunological, biochemical and molecular processes, which often results in critical illness (2). In addition to established risk factors such as age and the presence of certain comorbidities associated with poor COVID-19 prognosis and greater mortality risk (3), we recently identified that dysregulation of crucial biological pathways including acute inflammation, energy production, amino acid catabolism and lipid transport, among others, are associated with severe and critical disease (4). Accordingly, a better understanding of the role of biomolecules involved in these affected pathways might uncover novel predictive biomarkers or therapeutic targets for the treatment of immune dysregulation and complications driven by uncontrolled infection with severe acute respiratory syndrome coronavirus 2 (SARS-CoV-2). Indeed, multi-omics technologies have successfully identified potential biomarkers for the early prediction of severe COVID-19 outcomes (5), and both non-targeted and targeted metabolomics have been widely used to survey the metabolic changes occurring in patients with COVID-19 (6–15).

We recently found that severe COVID-19 outcomes were related to significant increases in serum glucose and glutamic acid levels in critically ill patients, suggestive of mitochondrial dysfunction (4). COVID-19 progression is associated with immune system hyperactivity that, in turn, is related to abnormal reprogramming of glucose metabolism (16). Along this line, recent studies have placed importance on mitochondrial health (17), as viruses are known to dysregulate mitochondria-associated mechanisms that participate in host innate immune responses to infection. In the context of COVID-19, changes to the levels of metabolites associated with mitochondrial dysfunction in patients might mirror perturbations of the immune system, making them potential surrogate markers of the disease stage. Some of these metabolites are known to have cytokine-like properties and signal through G protein-coupled receptors (GPCRs) (18). Two recent metabolomics studies reported increased levels of several energy-related signaling metabolites including succinate and α-ketoglutaric acid in patients with critical COVID-19 illness (19, 20). We therefore hypothesized that mitochondrial energy-related signaling metabolites implicated in host immune response to SARS-Cov-2 infection might serve as potential biomarkers for predicting higher risk of life-threatening illness.

Despite the great strides made in our understanding of COVID-19, there remains a need to identify and validate the accuracy of novel biomarkers in stratifying patients with the highest risk of developing critical COVID-19. To address this, we conducted a semi-targeted metabolomics analysis of 273 patients with different severity grades of COVID-19 recruited during the acute infection phase to determine the relative abundance of serum tricarboxylic acid (Krebs) cycle-related metabolites with known extracellular signaling properties (pyruvate, lactate, succinate and α-ketoglutarate). We then confirmed the significantly increased relative abundance of these metabolites by quantitative fluorimetric assays in patients with critical illness using a large validation cohort (n=398). Of the four metabolites, only pyruvate was an independent predictor of COVID-19 severity, and we used classification and regression tree (CART) analysis to calculate a cut-off value in serum for classifying patients with critical outcomes. The energy-related metabolites were positively associated with specific cytokines that are known biomarkers of COVID-19 severity, and with glucose levels, supporting the link between COVID-19 pathogenesis and immunometabolic dysregulation. Overall, our study highlights not only the key role of pyruvate, but also confirms its usefulness as a predictive biomarker in critically ill patients.

## Materials and methods

### Study design and participants

**Figure 1:**
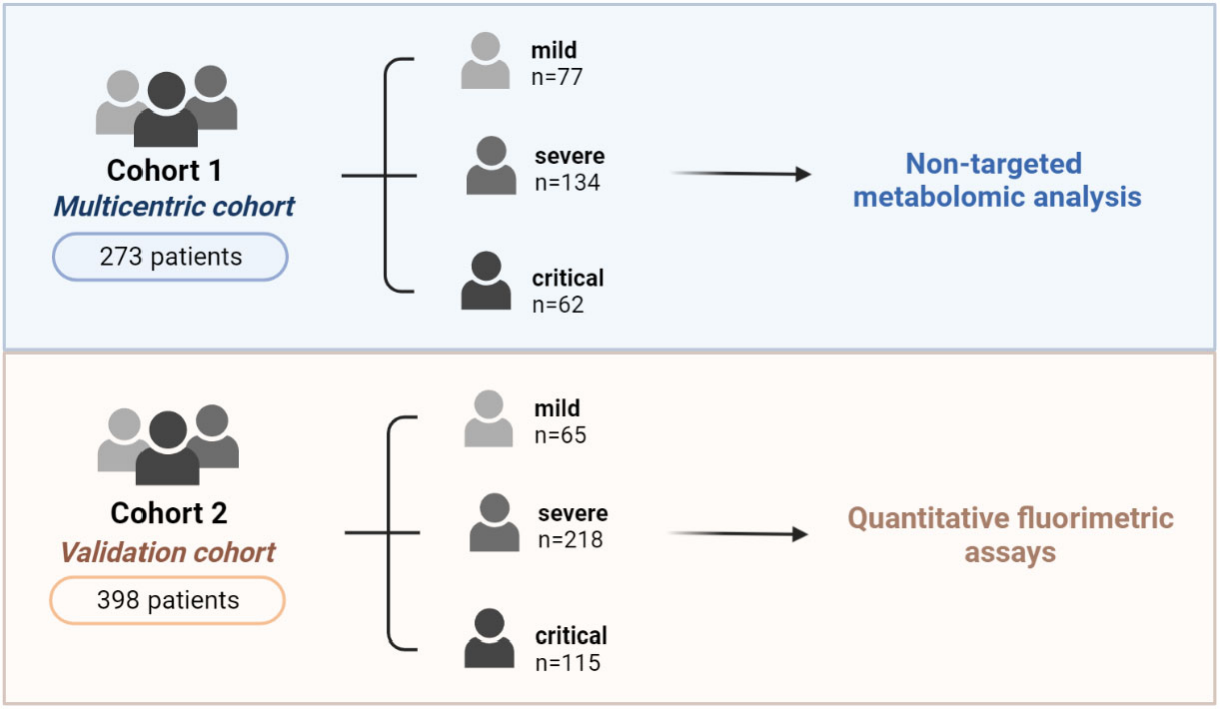
Study design and patient cohort distribution. Cohort 1 comprised 273 patients consecutively recruited during the first COVID-19 wave (March-May 2020) from the outpatient clinics of the participating hospitals. Cohort 2 included 398 patients with COVID-19 recruited from March 2020 to December 2020 at the HUJ23. Patients were stratified by disease severity. Serum samples were used for semi-targeted metabolomic analysis (cohort 1) and quantitative fluorimetric assays (cohort 2).

The present study included two cohorts of adult patients with SARS-CoV-2 infection confirmed by real-time reverse transcriptase-polymerase chain reaction assay (**Figure 1**). SARS-CoV-2 detection was carried out by RT-PCR following the guidelines from the Health Department of the Generalitat de Catalunya (21). Briefly, nasal and pharyngeal samples were collected using swabs into tubes containing transport medium for viruses, which were refrigerated at 4°C for a maximum of 48 hours until analysis. The RT-PCR technique was performed by cobas^©^ SARS-CoV-2 CE-FDA marking, with a sensitivity and specificity close to 100% (22, 23). Cohort 1 was a multicentric case-controlled cohort study of 273 patients with COVID-19 who were consecutively recruited during the first wave of the disease (March–May 2020) at outpatient clinics of the following hospitals: Hospital Universitari Joan XXIII (HUJ23), Tarragona; Hospital Virgen del Rocío, Sevilla; and Hospital Vall d’Hebrón, Barcelona. Cohort 2 included 398 patients with COVID-19 who were recruited from March 2020 to December 2020 at the HUJ23, and was used as a validation cohort. None of the patients enrolled in the present study got the SARS-CoV-2 vaccine at the time of blood sampling. For further validation of the results, 91 patients randomly selected from cohort 1 were also included in cohort 2. Patients enrolled in the study were stratified by disease severity (mild, severe or critical) according to the inclusion criteria described in “Diagnosis and Treatment Protocol for COVID-19 Patients (tentative 8^th^ edition)” (24). In brief: 1) *mild* patients were those who did not require hospitalization, with no or mild COVID-19 symptoms such as fever, dry cough, cephalea, asthenia, anosmia, diarrhoea, fatigue, myalgia, etc.; 2) *severe* patients were hospitalized patients presenting with fever, mild/moderate pneumonia, mild/moderate dyspnea, or patients with other pathologies developing severe symptoms in hospital; and 3) *critical* patients were hospitalized patients and/or that required intensive care due to severe pneumonia, respiratory failure, hemodynamic instability, tachypnea ≥30/min, O_2_ saturation ≤93%, PaO_2_/FiO_2_ ≤300, lung infiltrates ≥50% radiological fields in 24–48 h, septic shock, multi-organic dysfunction or failure. Patients’ characteristics are described in **Table 1** (cohort 1) and **2** (cohort 2).

**Table 1:** Patient characteristics of COVID-19 cohort 1.

COVID-19				
Variables	Mild (n = 77)	Severe (n = 134)	Critical (n = 62)	*P* value
**Gender (male)**	26 (33.8)	74 (55.2)	42 (67.7)	n.s.
**Age (years)**	41.0 (28.0-53.0)	64.0 (49.8-75.0)	65.0 (53.0-74.25)	<0.0001
**COVID-19-Mortality**	0 (0)	11 (8.2)	17 (27.4)	<0.0001
**Comorbidities**				
** Obesity**	6 (7.8)	23 (17.2)	12 (19.4)	<0.0001
** Metabolic syndrome**	0 (0)	9 (6.7)	8 (12.9)	<0.0001
** Diabetes mellitus**	2 (2.6)	24 (17.9)	18 (29.0)	<0.0001
** Hypertension**	7 (9.1)	60 (44.8)	30 (48.4)	<0.0001
** Cardiovascular disease**	4 (5.2)	31 (23.1)	6 (9.7)	<0.0001
**Inflammatory cytokines**				
** IL-7 (pg/mL)**	5.3 (1.6-10.1)	8.1 (3.4-11.8)	9.7 (4.6-20.4)	0.007
** IL-15 (pg/mL)**	6.7 (4.0-8.7)	10.9 (6.3-18.6)	15.1 (8.9-23.6)	0.001
** TNF-α (pg/mL)**	33.2 (18.1-60.0)	48.5 (34.3-81.7)	65.5 (53.7-87.8)	0.040
** IFN-γ (pg/mL)**	9.2 (4.1-16.5)	13.6 (4.9-24.0)	10.7 (5.4-23.6)	n.s.
**Biochemical parameters**				
** D-Dimer**	130.5 (99.3-198.5)	676.0 (398.0-1088.0)	752.0 (543.5-1394.5)	<0.0001
** Fibrinogen**	358.3 (430.5-521.5)	712.5 (563.8-826.0)	779.5 (640.0-923.5)	<0.0001
** IL-6**	12.8 (2.5-34.2)	13.6 (5.7-30.1)	29.2 (8.4-93.0)	n.s.
** Ferritin**	198.5 (84.0-502.8)	439.0 (172.0-1486.5)	770.0 (238.0-1571.0)	<0.0001
** CRP**	0.55 (0.1-3.8)	17.0 (6.1-53.1)	12.6 (5.6-17.7)	<0.0001
** Glucose**	91 (81.5-104.5)	110.0 (98.0-128.8)	118.0 (102.0-134.8)	<0.0001

Data are presented as n (%) and median (25^th^ and 75^th^ interquartile range) for qualitative and quantitative variables, respectively. IL, interleukin; TNF-α, tumor necrosis factor alpha; IFN- γ, interferon gamma; CRP, C-reactive protein. n.s., non-significant.

### Ethics

The study and all research protocols were approved by the Committee for Ethical Clinical Research by following the rules of Good Clinical Practice from the Institut d’Investigació Sanitària Pere Virgili (CEIm-IISPV, reference 079/2020). All participants gave written informed consent according to the Declaration of Helsinki. All information was collected and stored in a database specially designed for this purpose.

### General laboratory measurements

The sampling protocol included clinical evaluation, blood cell count, and standard biochemical parameters at inclusion. Serum samples were stored at -80°C at BioBank–Institut d’Investigació Sanitària Pere Virgili (IISPV) facilities until needed. Serum samples were collected within the first 21 days of the acute infection.

### Serum cytokine concentrations

Serum concentrations of selected molecules (IL-7, IL-15, TNF-α and IFN-γ) previously described in the inflammatory cascade of COVID-19 (25) were evaluated using a MILIPLEX assay (HSTCMAG-28SK-07, Millipore, Billerica, MA, USA), which was performed at the Center for Omic Sciences (COS), Reus, Spain.

### Semi-targeted metabolomics

Serum samples from patients in cohort 1 were evaluated using a semi-targeted metabolomics approach to measure the relative abundance of the following metabolites involved in energy metabolism: succinate, pyruvate, lactate and α-ketoglutarate. As previously described (4), the derivatized compounds from serum extraction were analyzed by time-of-flight gas chromatography (GC-qTOF) on an Agilent model 7200 (Agilent Technologies, Santa Clara, CA, USA) mass spectrometer. Relative abundance levels of metabolites were determined by EI-MS spectra and library retention time using the Fiehn 2013 Mass Spectral RTL Library. After putative identification, the compounds were semi-quantified in terms of the internal standard response ratio.

### Validation of targeted metabolites

Samples from cohort 2 were used to validate the semi-targeted metabolomic analysis. The quantification of selected metabolites was assessed by specific fluorometric assays in serum filtrates (10,000 kD). EnzyChrom™ Succinate, Pyruvate and L-Lactate assay kits were purchased from BioAssay Systems (Hayward, CA, USA). An assay kit for α-ketoglutarate was purchased from BioVision (Milpitas, CA, USA).

### Statistical analyses

Before the statistical analyses, the normal distribution and homogeneity of the variances were tested using the Kolmogorov-Smirnov test. Normally distributed data were expressed as the mean ± standard deviation (SD), whereas variables with a skewed distribution were represented as the median (25th percentile – 75th percentile). Categorical data were compared using the χ2 test, and continuous data were compared using the non-parametric Kruskal-Wallis test and Dunn’s multiple comparisons test for correction. Associations between quantitative variables were evaluated using Spearman correlation analysis. Regression analyses were employed (stepwise forward selection procedures) to identify the potential role of clinical and anthropometric variables as independent factors related to COVID-19 severity. Receiver operating characteristic (ROC) curves were constructed to assess the predictive value of independent factors for COVID-19 severity. In addition, we used classification and regression tree (CART) analysis, which splits the data into segments that are as homogenous as possible concerning the dependent variable. Statistical analyses were performed using SPSS (version 21.0, SPSS Inc., Chicago, IL), and graphical representations were generated with GraphPad Prism software (version 9.0, GraphPad Inc., San Diego, CA). The results were considered significant at P < 0.05.

## Results

### Multicentric cohort

#### Patients’ characteristics

Stratification of patients in cohort 1 (273 adults with SARS-CoV-2 infection) was as follows: mild (n=77), severe (n=134), and critical (n=62) (**Figure 1** and **Table 1**). As expected, COVID-19 severity was related to age, male sex and pre-existing medical conditions. The median age in the mild group was 41 years, patients were predominantly (ca. 70%) female, and there was no or a very low (<10%) prevalence of comorbidities. By contrast, the median age in the severe and critical groups was significantly higher (64 years), patients were predominantly male, and the prevalence of comorbidities such as obesity, metabolic syndrome, diabetes mellitus, hypertension and cardiovascular disorders was significantly greater. Also, the percentage of mortality directly related to COVID-19 was significantly greater in the critical group than in the moderate group of patients. Baseline levels of selected parameters previously related to an inflammatory state in acute COVID-19 were evaluated and, as expected, circulating concentrations of chemokines (IL-7, IL-15, TNF-α and IFN-γ) and biochemical parameters (D-dimer, fibrinogen, ferritin, C-reactive protein [CRP] and glucose) were all significantly higher in the severe and critical groups than in the mild group (**Table 1**).

#### Increased circulating energy-related metabolites in critical COVID-19

**Figure 2:**
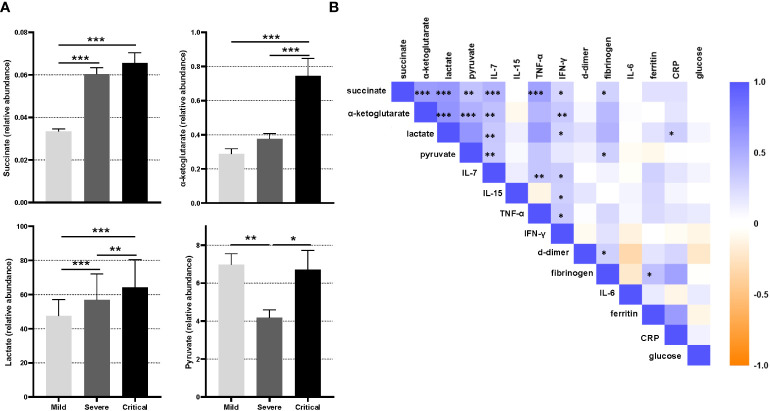
Semi-targeted metabolomics study of patients in cohort 1. **(A)** Relative abundance of succinate, α-ketoglutarate, lactate and pyruvate acid in patients grouped by disease severity (mild, severe and critical). Statistical significance between different groups was estimated using the Kruskal-Wallis test. Bars represent median values ± SEM. **(B)** Correlation matrix between relevant parameters previously related to COVID-19 severity and metabolites measured in patients of cohort 1. The color of the squares corresponds to the absolute value of the Spearman correlation coefficient, with orange or blue color indicating negative or positive correlation, respectively. A blank square indicates a lack of correlation between variables. The results were considered significant at *P<0.05; **P<0.01; ***P<0.001.

The relative abundance levels of succinate, pyruvate, lactate and α-ketoglutarate were evaluated in cohort 1 using semi-targeted metabolomics analysis. Circulating relative concentrations of succinate and lactate were significantly higher in severe and critical patients than in mild patients (**Figure 2A**, P<0.001). α-ketoglutarate concentrations were also significantly higher in critical patients than in both severe and mild patients (P<0.001). Likewise, circulating pyruvate concentrations were significantly higher in critical than in severe patients (P=0.013).

We next evaluated the relationship between succinate, pyruvate, lactate and α-ketoglutarate and the presence of comorbidities. α-ketoglutarate and lactate were significantly higher in patients with metabolic syndrome (P<0.001 and P=0.042, respectively), diabetes mellitus (P=0.006 and P=0.003, respectively) and hypertension (P=0.026 and P=0.005, respectively) than in peers without comorbidities. Patients with obesity showed increased α-ketoglutarate concentrations (P=0.036), and those with cardiovascular diseases presented higher succinate and lactate concentrations (P=0.032 and P=0.012, respectively). Notable, Spearman correlation analysis revealed a significant association between the selected parameters previously described in the inflammatory cascade of SARS-CoV-2 infection and the baseline circulating concentrations of the studied metabolites. Serum succinate, α-ketoglutarate, lactate and pyruvate levels were all positively associated with serum IL-7, IFN-γ and TNF-α (**Figure 2B**). Similarly, the four studied metabolites showed a mild correlation with fibrinogen, and only lactate showed a significant mild correlation with CRP (**Figure 2B**).

### Validation cohort

#### Patients’ characteristics

Study cohort 2 (398 adults with SARS-CoV-2 infection) was distributed as follows: mild (n=65), severe (n=218) and critical (n=115) (**Figure 1** and **Table 2**). Similar to the findings in cohort 1 (**Table 1**), the risk for progressing to severe illness increased with age, male sex and the presence of comorbidities such as obesity, metabolic syndrome, diabetes mellitus, hypertension and cardiovascular disease. Accordingly, the median age of patients with mild disease was lower (52 years), with a predominance of female gender (52.3%), and with a lower prevalence of comorbidities than in the severe and critical groups of patients. Conversely, in the critical group of patients, both the median age and the percentage of male sex was significantly higher (72 years and 70.4%, respectively). The percentage of mortality was also significantly higher in patients with the most critical COVID-19 outcomes (35.7%). As expected, the levels of the most relevant biochemical parameters (D-dimer, fibrinogen, ferritin, CRP, glucose and creatinine) were significantly higher in the severe and critical groups than in the mild group of patients.

**Table 2:** Patient characteristics of COVID-19 cohort 2.

Variables	COVID-19 group			*P* value
	Mild (n = 65)	Severe (n = 218)	Critical (n = 115)	
**Gender (male)**	31 (47.7)	135 (61.9)	81 (70.4)	<0.0001
**Age (years)**	52 (43.5-64.0)	59 (49.0-68.0)	72 (63.5-76.5)	<0.0001
**COVID-19-Mortality**	0 (0)	1 (0.5)	41 (35.7)	<0.0001
**Comorbidities**				
** Obesity**	11 (16.9)	64 (29.4)	29 (25.2)	<0.0001
** Metabolic syndrome**	4 (6.2)	15 (6.9)	20 (17.4)	<0.0001
** Diabetes mellitus**	9 (13.8)	34 (15.6)	32 (27.8)	<0.0001
** Hypertension**	21 (32.3)	83 (38.1)	60 (52.2)	<0.0001
** Cardiovascular disease**	7 (10.8)	19 (8.7)	15 (13.0)	<0.0001
**Biochemical parameters**				
** D-Dimer**	533.0 (339.8-937.3)	565.5 (397.0-849.8)	896.0 (577.8-1474.5)	<0.0001
** Fibrinogen**	641.0 (522.8-813.0)	765.0 (658.0-863.8)	784.0 (682.0-900.3)	<0.0001
** IL-6**	11.4 (4.2-44.9)	9.1 (3.0-19.7)	25.5 (8.2-73.6)	<0.0001
** Ferritin**	429.0 (232.0-637.0)	445.5 (257.8-860.0)	550.0 (371.0-1098.0)	n.s.
** CRP**	3.9 (1.0-8.2)	7.0 (3.5-11.5)	10.4 (4.8-16.9)	<0.0001
** Glucose**	106.0 (89.0-114.5)	104.0 (87.0-130.0)	122.0 (99.0-161.0)	<0.0001
** Creatinine**	0.8 (0.7-1.0)	0.8 (0.6-1.0)	0.9 (0.7-1.1)	0.008

Data are presented as n (%) and median (25^th^ and 75^th^ interquartile range) for qualitative and quantitative variables, respectively. IL, interleukin; CRP, C-reactive protein. n.s., non-significant.

#### Pyruvate as a potential biomarker of critical COVID-19

**Figure 3:**
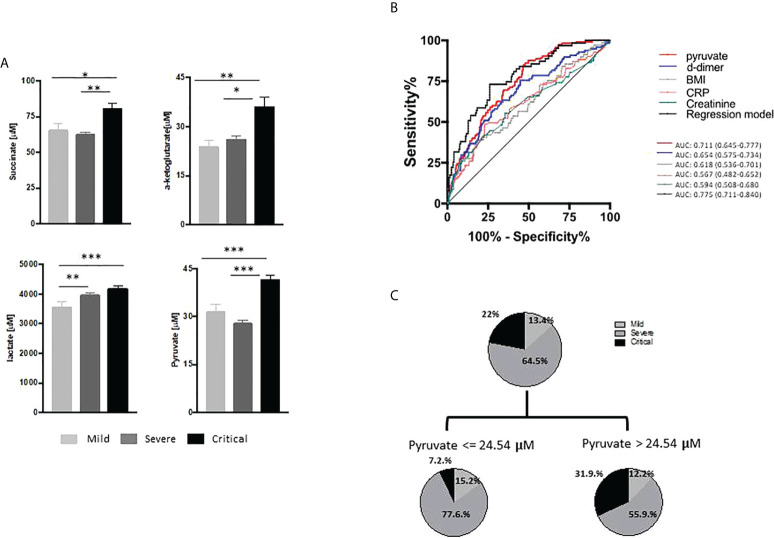
Energy-related metabolites in the validation cohort. **(A)** Serum levels (µM) of succinate, lactate, pyruvate and α-ketoglutarate determined by fluorimetric assay in patients with mild (n=65), severe (n=218) and critical (n=115) COVID-19 disease. Statistical significance was estimated using the Kruskal-Wallis test and Dunn’s multiple comparisons test. **(B)** Receiver operating characteristic curves predicting COVID-19 severity included the parameters of the regression model (**Table 3**). **(C)** Regression tree (CART) analysis including all the previously selected variables as predictors of COVID-19 severity. The results were considered significant at *P<0.05; **P<0.01; ***P<0.001.

To validate the increases in circulating succinate, pyruvate, lactate and α-ketoglutarate in critical COVID-19 patients, we measured their serum concentrations in cohort 2 using specific fluorometric assays. Circulating levels of succinate, pyruvate, lactate and α-ketoglutarate were confirmed to be higher in critical COVID-19 patients than in the mild and severe groups (**Figure 3A**).

We next used linear regression analysis to determine whether succinate, pyruvate, lactate and α-ketoglutarate were independent factors of COVID-19 severity. The four metabolites and the selected variables previously described as inflammatory or predictive biomarkers for critical COVID-19 outcomes were included in the model. The best regression model obtained showed that body mass index (BMI) (P=0.025) and circulating levels of creatinine (P=0.043), CRP (P=0.039), D-dimer (P<0.001) and pyruvate (P<0.001) were the main determinants of COVID-19 severity (**Table 3**). ROC curves were then constructed to assess the validity of the independent variables individually and to establish biomarkers for the prediction of COVID-19 severity (**Figure 3B**). Among all the independent variables, pyruvate showed the highest area under the ROC curve (AUC=0.711; 95% confidence interval [CI] 0.645–0.777). Moreover, the model provided an improved predictive capacity when considering all the variables together (AUC=0.775, 95% CI 0.711–0.840). To further confirm these results, we performed CART analysis including all the previously selected variables as predictors of COVID-19 severity (**Figure 3C**). According to this analysis, circulating pyruvate was the strongest predictor of COVID-19 severity (cut-off 24.50μM, P<0.001). Indeed, a high proportion (87%) of the critically ill patients showed circulating pyruvate concentrations >24.5μM and only nine (13%) had pyruvate levels <24.5μM (31.9% vs 7.2% of the total cohort).

**Table 3:** Clinical, analytical and anthropometric variables related to COVID-19 severity.

COVID-19 severity (R = 0.452; R^2 ^= 0.204)					
variable	β (non-standardized)	SE	95% CI	β (standardized)	P value
Constant	1.374	0.159	1.060-1.688	–	<0.001
Pyruvate	0.006	0.002	0.003-0.009	0.240	<0.001
D-dimer	8.29E-5	0.00002	0.000034-0.000132	0.216	<0.001
BMI	0.011	0.005	0.001-0.020	0.146	0.025
CRP	0.007	0.004	0.000377-0.014	0.134	0.039
Creatinine	0.183	0.090	0.006-0.361	0.132	0.043

Variables included in the regression model were body mass index (BMI), age and circulating levels of succinate, pyruvate, lactate, α-ketoglutarate, D-dimer, fibrinogen, Interleukin-6, ferritin, C-reactive protein (CRP), creatinine and glucose.

## Discussion

Our present study that included two different larger cohorts of 273 and 398 patients and used different techniques confirms a recent report showing an altered profile of circulating metabolites involved in glucose metabolism (pyruvate and lactate) and the Krebs cycle (α-ketoglutarate and succinate) in a small sample (n=65) of patients with severe and critical COVID-19 (20). Of note, we validated the levels of specific energy-related metabolites in COVID-19 disease using fluorimetric assays, which offer several advantages over metabolomics analysis, as it is a simple and economical yet quantitative method.

Mitochondrial dysfunction has been related to compromised immunity (26), and is also a hallmark of most COVID-19 risk factors including age, obesity, diabetes or cardiovascular disease (9, 20). Given that hypoxia is known to drive Krebs cycle reprogramming and is one of the main pathophysiologic features of COVID-19 severity, we postulated that circulating levels of energy-related metabolites might play a key role in predicting COVID-19 severity. Metabolomics approaches have been widely used to analyze the metabolic changes in patients with COVID-19 (6–8, 10, 11); however, alternative methods to omics-based techniques that provide reliable data without the need for expensive equipment and trained personnel could be crucial to validate the predictive potential of metabolites identified using semi-targeted analysis as biomarkers of COVID-19 outcomes. On the basis of the previous association between mitochondrial dysfunction and critical COVID-19 outcomes (4), the present manuscript specifically focused on these 4 metabolites with cytokine-like properties and signal through GPCRs to not only corroborate their involvement in the dysregulation of energy production in COVID-19, but also to assess their potential as signaling biomarkers for early detection of critical COVID-19 patients.

Metabolomics analysis of the primary cohort (semi-targeted approach in a multicentric study) revealed increased levels of pyruvate and lactate in critically ill patients. Pyruvate is the end-product of glycolysis and is a key intermediate that can be used by several metabolic pathways, including the Krebs cycle, which is known to be essential for viral infection and replication (27). Pyruvate can, however, be generated from other sources, including the oxidation of lactate by lactate dehydrogenase, of which high levels are associated with a higher risk of COVID-19 severity and mortality (28, 29). Lactate is an important end-product of anaerobic metabolism, and high lactate levels are considered an early sign of tissue hypoxia. Of note, in a study of 70 consecutive patients, Yang and collaborators reported that patients with severe disease did not have higher blood lactate values than peers with non-severe illness (30). Nonetheless, consistent with our results, most studies have demonstrated increased lactate levels in severe and non-survivor COVID-19 patients compared with patients with mild disease (31).

In addition to pyruvate and lactate, we also found increased levels of α-ketoglutarate and succinate in critical patients, which is consistent with the results of other studies showing elevated levels of the Krebs cycle intermediates malate and oxalacetate in critical patients, although citrate levels were decreased (4, 19, 20). Viral infections induce host inflammation, and we recently reported that succinate participates in immune activation, particularly in macrophage responses (32). Indeed, we and others demonstrated that succinate is generated as a part of an inflammatory program to promote a robust response during the acute phase of inflammation, while also acting as a pro-resolving signal to keep inflammation in check (33–36). Many inflammatory-related pathological conditions including metabolic diseases (37–40) are associated with elevated levels of circulating succinate. Similarly, metabolome studies have reported increased levels of succinate (41) and α-ketoglutarate (20) in patients with COVID-19. The chronic increase in these energy-related signaling metabolites mirrors perturbations of the immune system. Lactate and pyruvate might also participate in inflammatory programs, and both are elevated in some inflammatory-related conditions (42); pyruvate is known to have anti-inflammatory properties (43).

Quantification of metabolites by fluorometric assays in the validation cohort was consistent with the data of the semi-targeted metabolomic analysis. With the exception of pyruvate, all of the metabolites analyzed are signaling ligands for GPCRs (44, 45). GPCRs are the largest family of cell surface receptors and serve as pharmacological targets of ~34% of all FDA-approved drugs (41). In this context, Yang and colleagues hypothesized that GPR4, a pro-inflammatory receptor, may be implicated in the pathophysiology of COVID-19 and could be a potential therapeutic target to improve COVID-19-related complications (46). Although other intermediates of the Krebs cycle cannot be excluded, our present results in the context of signaling networks make lactate, succinate and α-ketoglutarate potential targets for the management of COVID-19 complications.

In addition to the altered profile of pyruvate, lactate, succinate and α-ketoglutarate in critical patients, we found positive correlations between energy-related metabolites and inflammatory cytokines, biomarkers of COVID-19 severity, and glucose levels, confirming the link between the pathogenesis of COVID-19 and the regulation of metabolism (9, 47, 48). Previous metabolomic-based studies have assessed the potential of circulating metabolites as diagnostic and prognostic biomarkers of COVID-19 (15, 49), however, none have confirmed their validity as quantitative biomarkers (including those variables known or likely to be associated with COVID-19 disease) using fluorimetric or colorimetric analysis, which allows for thresholds to be established for clinical practice. In the present study, we found that BMI, D-dimer, CRP, creatinine and pyruvate levels were the main determinants of COVID-19 severity. Similarly, elevated kynurenine levels, a metabolite of the amino acid tryptophan produced in response to immune activation and involved in many biological processes, were determined together with increased age, ferritine, creatinine and D-dimer, among others, as a promising blood biomarker to predict an increased risk of mortality in SARS-CoV-2 infected people (50). Herein, among all the variables, circulating pyruvate levels better predicted the progression of the disease. Pyruvate alone and the regression model including the aforementioned variables had similar AUC values, and CART analysis confirmed these results, with pyruvate being the unique node in the decision tree. Previous metabolomic studies have included pyruvate in a panel of circulating biomarkers and metabolites for COVID-19 diagnosis and prognosis (15, 49). Interestingly, in a subsample of the two cohorts (n=91), we found a positive correlation between circulating pyruvate levels measured by metabolomic and measured by fluorimetric analysis (R=0.551; P<0.001). With regards to the role of pyruvate in SARS-CoV-2 pathophysiology, it might have an anti-inflammatory function in macrophage activation, similar to the effect observed during influenza A virus infection (43), and it might also provide therapeutic benefit by suppressing viral replication through Krebs cycle induction (51).

In conclusion, we propose the fluorimetric quantification of circulating pyruvate as a cost-effective clinical decision support tool for the early classification of critical patients, which could be easily implemented in daily clinical practice as part of the inflammatory profile of routine blood analyses. Notwithstanding, further studies will be needed to elucidate the role of pyruvate and other potential energy-related metabolites in the pathophysiology of COVID-19. Thereby, to depict the entire landscape of factors contributing to the pathophysiological changes associated with COVID-19, other potential influencing elements than those directly linked to metabolic dysregulation, such as hypoxemia induced in patients with severe pneumonia, might also be considered. Overall, our results may help to improve patient stratification in COVID-19, providing early diagnosis tools to identify high-risk subgroups.

## Group members

The COVIDOMICS Study Group from Hospital Universitari Joan XXIII/IISPV/URV is composed of the following investigators who should be considered contributors to the paper: Sonia Espineira, Elena Yeregui, Jenifer Masip, Verónica Alba, Montserrat Vargas, Anna Martí, Consuelo Viladés, Frederic Gómez-Bertomeu, Graciano García-Pardo, Montserrat Olona, Laia Bertrán, Carmen Aguilar, José Antonio Porras, Sergi Veloso, Ajla Alibalic, David Riesco, Mónica Real, Jessica Binetti, Judit Poblet, Mercé Sirisi, Gaspar Dalmau, Vanessa Gázquez, Esther Rodriguez, Antonia Garcia, Esther Picó, Cristina Gutiérrez, Gemma Recio-Comí, Carla Martin-Grau, Teresa Sans, Carlos Chiapella, Pilar Carbajo, Mireia Cramp, Carme Bes, Rosalia Bote, Nuria Alba, Blanca Rosich, Cristina Varillas, Catherine Cabrejo, Júlia Vidal-González, Rosaura Reig, Llorenç Mairal, Jesús Esteve Ferrán, Neus Camañes, Angela Cortés and Rafael Gracia.

## Data availability statement

The raw data supporting the conclusions of this article will be made available by the authors, without undue reservation.

## Ethics statement

The studies involving human participants were reviewed and approved by Committee for Ethical Clinical Research by following the rules of Good Clinical Practice from the Institut d’Investigació Sanitària Pere Virgili (CEIm-IISPV, reference 079/2020). The patients/participants provided their written informed consent to participate in this study.

## Author contributions

VC-M. and LR participated in the conception and design of the study, did the statistical analysis and wrote the manuscript. JP, AG-V, MB, RJ, TA and MO participated in the recruitment of participants and sample procurement. AM and EM-M performed quantification of metabolites by fluorometric assays. ML-D, ER-M, JV and FV provided scientific discussion and revised the manuscript. AR and SF-V conceived and supervised this study and wrote the manuscript. All authors read and approved the final version of the manuscript.

## Funding

This work has been developed in the framework of the COVIDOMICS’ project supported by Direcció General de Recerca i Innovació en Salut (DGRIS), Departament de Salut, Generalitat de Catalunya (PoC-6-17 and PoC1-5). The research was also funded by the Programa de Suport als Grups de Recerca AGAUR (2017SGR948), the SPANISH AIDS Research Network [RD16/0025/0006]-ISCIII-FEDER (Spain) and the Centro de Investigación Biomédica en Red de Enfermedades Infecciosas-ISCIII [CB21/13/00020], Madrid, Spain. LR is supported by the Instituto de Salud Carlos III (ISCIII) under grant agreement “CD20/00105” through the program “Contratos Sara Borrell”. FV is supported by grants from the Programa de Intensificación de Investigadores (INT20/00031)-ISCIII and by “Premi a la Trajectòria Investigadora dels Hospitals de l’ICS 2018”. AR is supported by a grant from IISPV through the project “2019/IISPV/05” (Boosting Young Talent), by GeSIDA through the “III Premio para Jóvenes Investigadores 2019” and by the Instituto de Salud Carlos III (ISCIII) under grant agreement “CP19/00146” through the Miguel Servet Program. This study was also supported by grants SAF2015–65019-R and RTI2018–093919-B-100 (to SF-V.) funded by MCIN/AEI and by “ERFD A way of making Europe”; PI19/01337 to FV, PI20/00095 to VC.-M, PI20/00326 to AR and PI20/00338 to JV funded by ISCIII, cofinanced by the European Regional Development Fund (ERDF), and from Fundación Bancaria Caixa d’Estalvis i Pensions de Barcelona (HR20-00051 to SF-V). The Spanish Biomedical Research Center in Diabetes and Associated Metabolic Disorders (CIBERDEM) (CB07708/0012) is an initiative of the Instituto de Salud Carlos III. SF-V acknowledges support from the Miguel Servet tenure-track program (CP10/00438 and CPII16/00008) from the Fondo de Investigación Sanitaria, cofinanced by the ERDF. VC-M acknowledges support from the Ramón y Cajal program (RYC2019-026490-I) from the Spanish Ministry of Science and Innovation, cofinanced by the ERDF. The work was also supported by Consejeria de Salud y Familia (COVID-0005-2020), Consejeria de Transformacion Economica, Industria, Conocimiento y Universidades Junta de Andalucia (CV20-85418to ER-M) and Instituto de Salud Carlos III (ISCIII) under grant agreement CP19/00159 to AGV “a way to make Europe”. ER-M was supported by the Spanish Research Council (CSIC). The funders have no roles in study design, data collection, data analysis, interpretation or the writing of this research.

## Acknowledgments

This study would not have been possible without the generous collaboration of all the patients and their families and medical and nursing staff who have taken part in the project. We particularly acknowledge the collaboration of the Departments of Preventive Medicine and Epidemiology, Internal Medicine, Critical Care, Emergency, Occupational Health, Laboratory Medicine and Molecular Biology, and BioBank-IISPV (B.0000853 + B.0000854) integrated into the Spanish National Biobanks Platform (PT20/00197), CERCA Program (Generalitat de Catalunya) and IISPV, for their collaboration. We also thank Pol Herrero, Maria Guirro and Antoni del Pino from the Proteomics and Metabolomics facilities of the Centre for Omic Sciences (COS) Joint Unit of the Universitat Rovira i Virgili-Eurecat for their contribution to mass spectrometry analyses.

## Conflict of interest

The authors declare that the research was conducted in the absence of any commercial or financial relationships that could be construed as a potential conflict of interest.

## Publisher’s note

All claims expressed in this article are solely those of the authors and do not necessarily represent those of their affiliated organizations, or those of the publisher, the editors and the reviewers. Any product that may be evaluated in this article, or claim that may be made by its manufacturer, is not guaranteed or endorsed by the publisher.
